# Towards accurate and precise *T*_1_ and extracellular volume mapping in the myocardium: a guide to current pitfalls and their solutions

**DOI:** 10.1007/s10334-017-0631-2

**Published:** 2017-06-12

**Authors:** Donnie Cameron, Vassilios S. Vassiliou, David M. Higgins, Peter D. Gatehouse

**Affiliations:** 1 Norwich Medical School, University of East Anglia, Bob Champion Research and Education Building, James Watson Road, Norwich, NR4 7UQ UK; 2 Royal Brompton Hospital and Imperial College London, Sydney Street, London, SW3 6NP UK; 3 Philips Healthcare, Guildford Business Park, Guildford, Surrey, GU2 8XG UK

**Keywords:** *T*_1_ mapping, Accuracy, Precision, Cardiovascular magnetic resonance, Extracellular volume

## Abstract

Mapping of the longitudinal relaxation time (*T*_1_) and extracellular volume (ECV) offers a means of identifying pathological changes in myocardial tissue, including diffuse changes that may be invisible to existing *T*_1_-weighted methods. This technique has recently shown strong clinical utility for pathologies such as Anderson-Fabry disease and amyloidosis and has generated clinical interest as a possible means of detecting small changes in diffuse fibrosis; however, scatter in *T*_1_ and ECV estimates offers challenges for detecting these changes, and bias limits comparisons between sites and vendors. There are several technical and physiological pitfalls that influence the accuracy (bias) and precision (repeatability) of *T*_1_ and ECV mapping methods. The goal of this review is to describe the most significant of these, and detail current solutions, in order to aid scientists and clinicians to maximise the utility of *T*_1_ mapping in their clinical or research setting. A detailed summary of technical and physiological factors, issues relating to contrast agents, and specific disease-related issues is provided, along with some considerations on the future directions of the field.

## Introduction

Mapping of the longitudinal relaxation time, *T*_1_, and extracellular volume (ECV) in the human heart has recently shot to prominence on the merits of the modified Look–Locker inversion recovery (MOLLI) imaging sequence and related techniques [1]. These methods allow quantitative tissue characterisation in the myocardium, adding new information to that provided by *T*_1_-weighted techniques such as late gadolinium enhancement (LGE) imaging. For focal fibrosis, LGE provides excellent delineation of lesions with some means of quantifying their volume; however, LGE does not give a *T*_1_ estimate and may not be able to identify widely distributed or diffuse myocardial diseases. For example, when LGE is applied in diffuse fibrosis, the myocardium can appear isointense and indistinguishable from normal myocardium, as multiple uncalibrated factors affect the image brightness. These are clear limitations of LGE techniques, and in such situations quantitative *T*_1_ and ECV mapping is advocated [2, 3]. Use of myocardial *T*_1_ mapping is now widespread, with most MRI manufacturers offering *T*_1_ mapping solutions. However, great care must be taken when applying these methods clinically, given the need for protocol optimisation and locally-derived normal ranges.

The accuracy and precision of myocardial *T*_1_ mapping has been the focus of several studies to date [4–7], and has been discussed to some degree in much of the literature. Innovations in the field are considered in terms of their impact on accuracy and precision, typically offering trade-offs in one or the other for faster or more-accommodating scans. However, there are still several longstanding pitfalls associated with *T*_1_ and ECV mapping that affect accuracy and precision, and this review aims to give a comprehensive description of these, along with potential solutions, with the intent of aiding physicists and clinicians to maximise the clinical utility of *T*_1_ mapping. Indications will also be given as to what is reasonably achievable with myocardial *T*_1_ mapping in specific clinical applications, without a full clinical review, for which the reader is directed to Haaf et al. [8], Taylor et al. [9], Kammerlander et al. [10], and Schelbert and Messroghli [11], amongst others. The fundamentals of *T*_1_ mapping methods, the available pulse sequences, and the history of the technique will be discussed briefly, but again readers are directed to more-detailed reviews for full technical information: for example, by Kellman et al. [5] and Higgins and Moon [12]. Finally, the consensus statement of the Society for Cardiovascular Magnetic Resonance (SCMR) and the CMR Working Group of the European Society of Cardiology can be consulted for recommendations on how to set up a robust *T*_1_ mapping protocol [13]. A follow-up parametric mapping consensus statement from the SCMR and the European Association for Cardiovascular Imaging (EACVI) is in preparation.

This review will focus on sources of bias and variability in myocardial *T*_1_ and ECV estimation: first, technical and physiological pitfalls; second, issues relating to contrast agents; and third, specific disease-related issues. It will conclude with future directions of the field and some summary recommendations, including guidance on how *T*_1_ estimation accuracy might be traded for increased clinical utility. Within each section, pitfalls will be introduced, their mechanisms described, and suggestions offered for how to mitigate or eliminate them, along with possible future solutions, where available. For ease of reference, Table 1 lists the technical and physiological pitfalls discussed in this review in the order they are introduced in the text, with summaries of their relative effects on *T*_1_ and ECV mapping accuracy and precision.

**Table 1:** A summary of technical and physiological pitfalls and their effects on accuracy and precision in *T*_1_ and ECV mapping

Pitfall	Mechanism	Effect on *T*_1_ mapping		Effect on ECV mapping		Solutions (developing or speculative)
		Acc.	Prec.	Acc.	Prec.	
Look–Locker correction	Assumes continuous, low-flip-angle spoiled GRE; MOLLI *T*_1_ mapping can violate these assumptions	••	•	••	•	Pauses in seconds, saturation recovery sequence, shallower flip angle
Partial volume	Coarse in-plane resolution leads to inclusion of multiple tissues in some voxels, causing *T*_1_ errors	••	••	••	••	Conservative ROI drawing, finer in-plane resolution, (black-blood mapping, slower segmented acquisition)
Prep. pulse factors	Adiabatic prep. pulses, robust to *B*_0_ and *B*_1_ inhomogeneity, are long and lead to *T*_2_ decay and *T*_1_ bias	•••	••	•	•	Appropriate pulse design and/or optimised *B*_0_ and *B*_1_ shimming
Multishot bSSFP readout	Sensitive to *T*_2_ and off-resonance and requires catalysation before each imaging readout	•••	••	•	•	Appropriate catalysation scheme, coarser in-plane-resolution, shorter TR. Alternatively, use spoiled GRE
Field strength	*B*_0_ and *B*_1_ inhomogeneity ↑ with field strength, *T*_1_↑, *T*_2_↓, SNR↑	••	•	•	•	Appropriate *B*_0_ and RF shimming, trading off SNR for shallower flip angle, longer pause intervals
*k*-space filling	Linear ordering causes saturation, centric ordering leads to artefacts	•	•	•	•	Linear ordering is adequate, (paired ordering may offer benefits)
Signal-to-noise	Noise level affects sampled *T*_1_-weighted data when fitting	••	••	••	•	Consider thicker slices, coarser in-plane resolution, using a 3T system
Poor breath-holding	Misregistration of source images and *T*_1_ error	••	••	••	••	Patient coaching, image registration, (free-breathing *T*_1_ mapping)
Cardiac motion	Mistimed acquisitions and misregistration of images	••	••	••	••	Fit acquisition into diastolic/systolic pause, image registration
Flowing blood	Mixture of magnetisation histories in left-ventricular blood	•	•	•	•	Pauses in seconds; position patient carefully or avoid short bore MRI systems, if possible
Magnetisation transfer (MT)	Exchange between free and bound water pools distorts *T*_1_ recovery, causing *T*_1_ underestimation	••	•	•	•	Lower flip angle, longer TR, saturation recovery sequence. (Alternatively, MT may improve sensitivity to disease)

This table provides an overview of the pitfalls listed in the text, with no intended significance in the order they appear. The pitfalls are listed with the mechanisms by which they influence *T*_1_ and ECV mapping, qualitative ratings of how they affect *T*_1_ and ECV accuracy and precision, and possible strategies for eliminating or mitigating them, with developing or speculative solutions shown in brackets. Given the popularity of the original modified Look–Locker inversion recovery (MOLLI) *T*_1_ mapping sequence, it is considered the standard here, with other sequences being offered as potential solutions to pitfalls. Ratings are given as one to three blots, with • being mild, •• being moderate, and ••• being severe; however, it should be clear that none of the pitfalls dominate, and their relative importance is highly dependent on the application

*ECV* extracellular volume, *GRE* gradient recalled echo, *RF* radiofrequency, *ROI* region of interest, *SNR* signal-to-noise ratio, *TR* repetition time, and *bSSFP* balanced steady-state free-precession

### A brief introduction to *T*_1_ mapping methodology

All routinely available *T*_1_ mapping methods currently rely on preparing the longitudinal magnetisation using inversion or saturation radiofrequency (RF) pulses, applied to the whole imaging volume. Many pulse sequences for *T*_1_ mapping can be grouped according to the magnetisation preparation: inversion recovery sequences, including MOLLI [1, 14] and shortened MOLLI (ShMOLLI) [15]; saturation recovery sequences, including independent saturation recovery single-shot acquisitions (SASHA) [16], saturation method using adaptive recovery times for cardiac *T*_1_ mapping (SMAR*T*_1_Map) [17], and short acquisition period *T*_1_ (SAP-*T*_1_) [18]; and mixed preparation sequences, such as saturation pulse-prepared heart-rate independent inversion-recovery (SAPPHIRE) [19]. In practice, a mixture of magnetisation preparations and *T*_1_-weighted image acquisitions are performed during a breath-hold, over several cardiac cycles. The aim is to sample the *T*_1_ recovery over a range of delay times, and pixel-by-pixel curve-fitting is used to estimate *T*_1_ values. This produces a pixelwise *T*_1_ map, often with other output maps as quality indicators; see Kellman et al. for a flowchart illustrating the pipeline of *T*_1_ and ECV map generation [20]. Most MRI manufacturers provide in-line software for map calculation, but open source tools are also available [21, 22].

Non-mapping approaches can also estimate *T*_1_ in the heart: the inversion-recovery cine sequence, also known as the Look–Locker cine (LL-cine) technique [23, 24], relies on averaged signal intensities over regions of interest (ROIs) for *T*_1_ curve-fitting. This approach uses spoiled gradient recalled echo (GRE) cine, avoiding the factors affecting single-shot balanced steady-state free-precession (bSSFP). Note that many studies that have used the LL-cine method have repeated it at multiple washout times, as this improves ECV accuracy [24].

Given its popularity, the original MOLLI sequence will be considered the default method in this review, with other *T*_1_ mapping methods and schemes being addressed where appropriate. A common nomenclature for *T*_1_ mapping schemes will also be adopted [5]. This notation lists the number of images acquired following a magnetisation preparation pulse, along with the free-recovery, inter-inversion pause in brackets. Timings are given in beats, “b”, or seconds, “s”. For example, 3b(3b)3b(3b)5b uses three Look–Locker sets, of three beats, three beats, and five beats, respectively, with pauses of three R–R intervals between each set. A 5s(3s)3s scheme uses two Look–Locker sets and a minimum pause of 3 s between these, rounded up to the next whole R–R interval.

## Technical and physiological sources of error

### Look–Locker correction

The original 3b(3b)3b(3b)5b MOLLI sequence [1] and subsequent optimised versions rely on Look and Locker’s correction for rapid *T*_1_ estimation: namely, for sampling of the recovering longitudinal magnetisation with a series of small-flip-angle excitation pulses [25, 26]. The magnetisation is perturbed by these excitation pulses, causing flattening of the recovery curve, and yielding an apparent *T*_1_, known as *T*_1_*, when curve fitting:

1 $$S(t) = A - B\exp ({{ - TI} \mathord{\left/ {\vphantom {{ - TI} {T_{1}^{*} }}} \right. \kern-0pt} {T_{1}^{*} }}),$$

where *S*(*t*) is the signal at time TI after inversion. The “true” *T*_1_ is usually longer than *T*_1_* and can be calculated using the so-called Look–Locker correction:

2 $$T_{1} \approx T_{1}^{*} ({B \mathord{\left/ {\vphantom {B A}} \right. \kern-0pt} A} - 1).$$

The Look–Locker correction assumes continual repetition of a small flip angle spoiled GRE readout. MOLLI-based techniques violate the Look–Locker assumptions by using: (1) a bSSFP readout, which is sensitive to *T*_2_ and, weakly, to magnetisation transfer (MT), unlike spoiled GRE; (2) a relatively large excitation flip angle, 35° at 1.5T; and (3) an intermittent sampling scheme, governed by heart rate for original MOLLI, to maximise the spread of inversion recovery times. Factors (1) and (2) lead to progressive saturation of the longitudinal magnetisation, causing a negative *T*_1_ bias even after Look–Locker correction. Furthermore, Look–Locker sets are separated by pause intervals, aiming to allow sufficient recovery of the magnetisation prior to the next inversion pulse. If any of these pause intervals are too short for the application, be it native or post-contrast, this can lead to additional bias in *T*_1_ estimates. This can be particularly problematic for fast heart rates, as well as longer *T*_1_ values, such as in native myocardium at 3T.

Bias resulting from limited magnetisation recovery can be mitigated by using an optimised MOLLI acquisition scheme: by extending the inter-inversion pause in beats or by specifying pauses in seconds rather than beats [4]. The 2013 SCMR consensus recommends a 5s(3s)3s scheme for native *T*_1_ mapping and a 4s(1s)2s(1s)1s scheme for post-contrast acquisitions [13].

Alternatively, saturation recovery methods such as SASHA [16] sample the recovering longitudinal magnetisation using an independent preparation in each heartbeat, obviating the need for Look–Locker correction over multiple shots at the expense of a loss in precision due to a smaller dynamic range of *T*_1_ recovery. The saturation recovery is still affected during each SASHA single-shot readout, but this produces negligible bias in *T*_1_ estimates obtained from curve fitting of the independent saturation-recovery images [16, 27].

### Partial volume

Partial volume of different tissues within a voxel is prevalent in *T*_1_ mapping with single-shot imaging, where in-plane spatial resolution is necessarily somewhat coarse. Furthermore, the endo- and epi-cardial borders of the myocardium are often oblique to the imaged slice, especially for planes far from the mid-ventricle or in abnormal ventricles. Partial volume can lead to substantial errors, which motivates careful ROI delineation on *T*_1_ maps [28].

Partial volume causes bias in the apparent *T*_1_, especially when the tissues included in a voxel have strongly different *T*_1_ values. Prominent effects occur at the endocardial border where the difference in *T*_1_ between blood and myocardium leads to overestimation of native *T*_1_ estimation in subendocardial voxels. Partial volume also occurs between myocardium and other tissues, most often fat, whose chemical shift causes variable bias in the pixel *T*_1_ [29]; this has clinical relevance, and is discussed further in the “Errors in specific clinical applications” section.

Finer in-plane resolution of the *T*_1_ mapping sequence would theoretically reduce partial volume effects, but demands longer single-shot imaging duration if no trade-offs are made, increasing the risk of cardiac-motion blurring. Parallel imaging and partial Fourier in the phase-encode direction are commonly employed to allay this problem. It is essential to avoid partial volume when drawing regions of interest (ROIs), which are often limited to mid-wall when possible [28, 30]. Cardiac motion during the single-shot imaging also corrupts myocardial signal with blood signal in less obvious ways, and is discussed later.

“Black-blood” *T*_1_ mapping aims to eliminate blood partial volume for improved *T*_1_ estimation accuracy [31], whereby the magnetisation of flowing blood is nulled by motion-sensitive dephasing immediately before each single-shot image. Multiecho fat-water-separated imaging strives to separate fat signal from the thin RV [32], and has been combined with the same method of blood suppression [33]. However, these approaches are not routinely reliable.

To achieve finer spatial resolution, and thus reduce the impact of partial volume in source images on pixelwise mapping, segmented *k*-space image acquisition over multiple cycles is required, and is often combined with some undersampling strategy [34, 35]. Segmented acquisition is severely affected by R–R variability in inversion-recovery methods, and is very slow to acquire the fully recovered image in saturation-recovery methods. The non-mapping LL-cine approach can acquire fine spatial and temporal resolutions, but cardiac motion occurs during recovery, raising questions about the impact of through-slice motion, and substantial post-processing labour is required to optimise ROIs used for curve-fitting and regional estimation of *T*_1_.

Finally, free-breathing *T*_1_ mapping methods promise to reduce intershot-motion-related errors [36–39]. However, these methods are currently time-consuming.

### Factors affecting magnetisation preparation pulses (*B*_*1*_, *B*_0_, *T*_2_)

The fitting models used in *T*_1_ mapping usually assume exact inversion or saturation of the longitudinal magnetisation, or fit an extra parameter instead, reducing precision. Another approach uses prior knowledge of an inversion factor, which can be estimated from fully relaxed reference images to correct the estimated *T*_1_ [40–42].

**Fig. 1:**
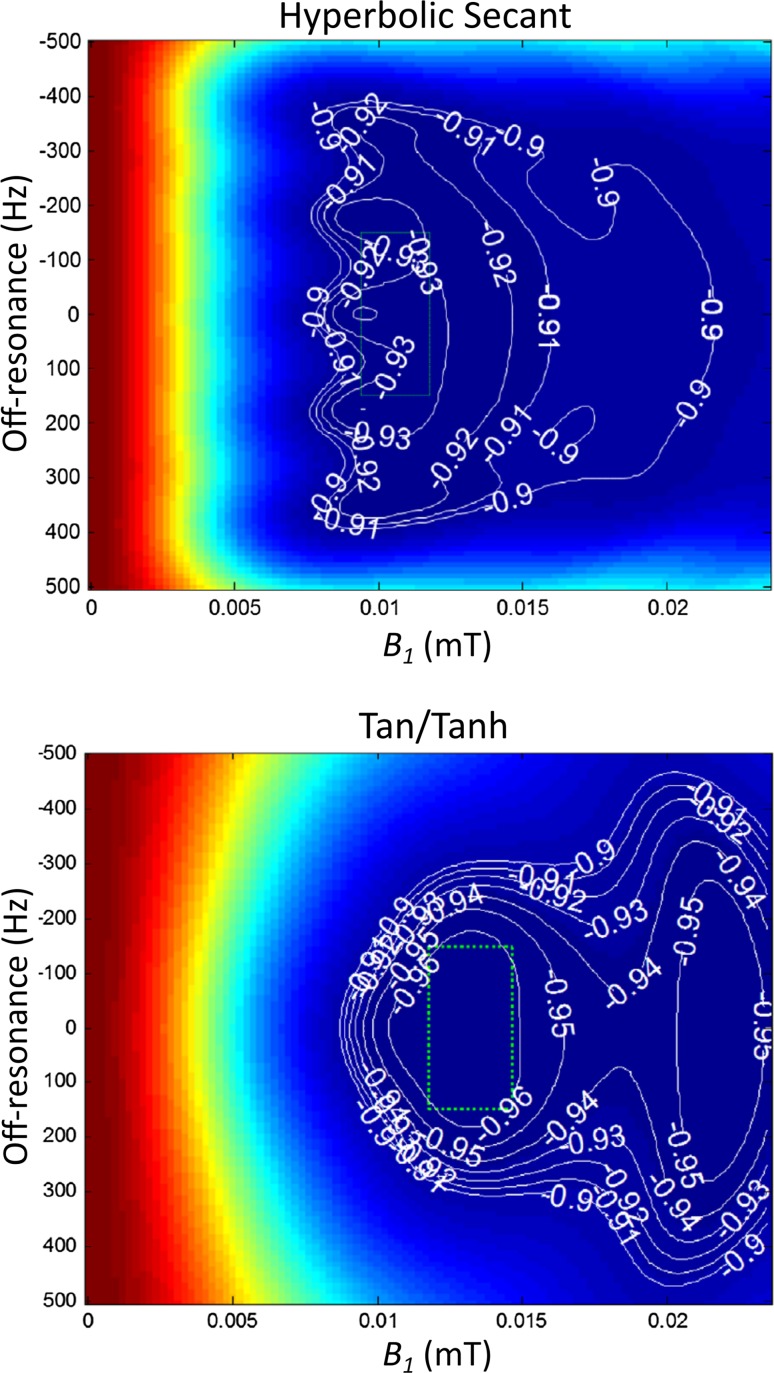
Inversion pulse performance. *Plots* show the response to *B*_0_ (*vertical*) and *B*_1_ (*horizontal*) of two different adiabatic inversion pulse designs: Hyperbolic Secant and Tan/Tanh (*T*_1_ = 1000 ms, *T*_2_ = 45 ms). A contour value −1.0 would indicate perfect inversion. The “design region”, enclosing the maximal likely in vivo cardiac *B*_0_ and *B*_1_ distortion, is represented by the *dotted green box*. Adapted with permission from Kellman et al. [40]

Conventional RF pulses require accurate RF transmit (*B*_1_) fields to achieve their prescribed flip-angle, so are sensitive to *B*_1_ inhomogeneity, which can be substantial across the heart, particularly at 3T [40, 43]. The flip-angle achieved by conventional non-selective RF pulses is also affected by off-resonance errors due to *B*_0_ inhomogeneity. Therefore, adiabatic inversion pulses or composite saturation pulses are widely used for mapping to reduce sensitivity to both *B*_0_ and *B*_1_ inhomogeneity [40, 44]. However, the longer duration of some adiabatic pulses can increase *T*_2_ decay during their execution and introduce sensitivity to off-resonance phase accumulation. Kellman, Herzka, and Hansen suggest the use of a relatively short tan/tanh adiabatic pulse for optimal inversion efficiency [40] (Fig. 1), while a composite saturation design is recommended for saturation-recovery methods such as SASHA, provided the higher specific absorption rate and pulse duration are acceptable [44]. Optimised saturation precision, or efficiency, is important in SASHA for two reasons: (1) to enable two-parameter curve-fitting for *T*_1_ estimation, as opposed to fitting of saturation efficiency as a third parameter; and (2), reliable removal of any history effect from previous cycles by nulling the longitudinal magnetisation prior to each independent shot.

**Fig. 2:**
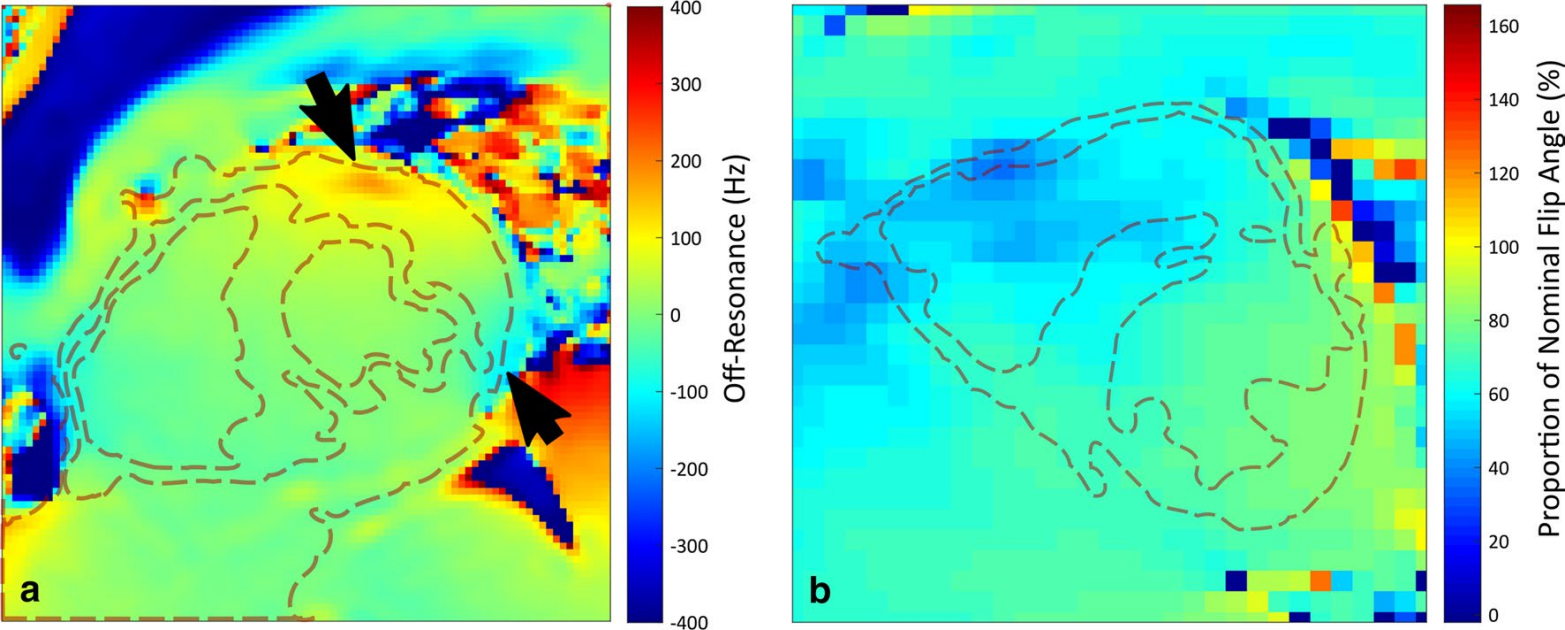
In vivo fieldmaps of static field and radiofrequency transmit homogeneity in the heart. *Plots* show a *B*_0_ map (**a**) and a *B*_1_ map (**b**) planned across the mid-ventricular short axis of the heart, with *dashed red lines* delineating the approximate boundaries of the myocardium. Maps were acquired at 3T, subsequent to first-order *B*_0_ shimming and dual-channel RF calibration. Note the distinct inhomogeneity of *B*_0_ near the coronary veins in a (*arrows*), and slightly reduced pulse performance across the right ventricle in **b**, where the measured *B*_1_ drops to around 50–60% of the nominal value

Although the aforementioned RF pulse designs are more tolerant to *B*_0_ and *B*_1_ errors, for mapping it is vital to optimise both *B*_0_ and *B*_1_ over the relevant volume. See Fig. 2 for examples of *B*_0_ and *B*_1_ maps, in vivo. The advent of RF (*B*_1_) shimming hardware with optimised volume calibration methods has enabled substantial improvements in *B*_1_ uniformity over the heart [45] even with only two whole-body transmitter channels [46].

### Inversion-recovery multishot bSSFP

Most *T*_1_ mapping methods use bSSFP to sample the recovering longitudinal magnetisation, as this method offers a higher signal-to-noise ratio (SNR) than spoiled GRE. However, bSSFP is sensitive to *T*_2_ and off-resonance, where both sensitivities are modulated by excitation flip-angle, RF pulse repetition time (TR), and magnetisation transfer [47]. These factors have greater impact on estimated *T*_1_ when the bSSFP flip angle is higher or the TR is longer, and native myocardial *T*_1_ values are more affected than the shorter post-contrast *T*_1_ values. Off-resonance is larger at 3T, mitigated by use of a lower flip angle [48], but reducing *T*_1_ error through use of a shorter TR is largely limited by patient peripheral nerve stimulation [49].

Another consideration with bSSFP is the transient period before the steady-state is established, which is characterised by oscillatory magnetisation, causing image artefacts. The intensity of the oscillations depends on both the bSSFP catalysation used to stabilise the signal, and the *k*-space trajectory [50].

#### Catalysation sequences for bSSFP

For *T*_1_ mapping, some single-shot bSSFP images at short inversion-recovery times are required for optimal curve-fitting, although for SASHA a later start is preferred, as earlier readouts have low SNR [27]. Short delay times prevent stabilisation of the bSSFP signal before phase-encoded data acquisition commences, and since all shots should be acquired with identical parameters, a longer stabilisation for the later inversion-recovery shots is inadvisable.

**Fig. 3:**
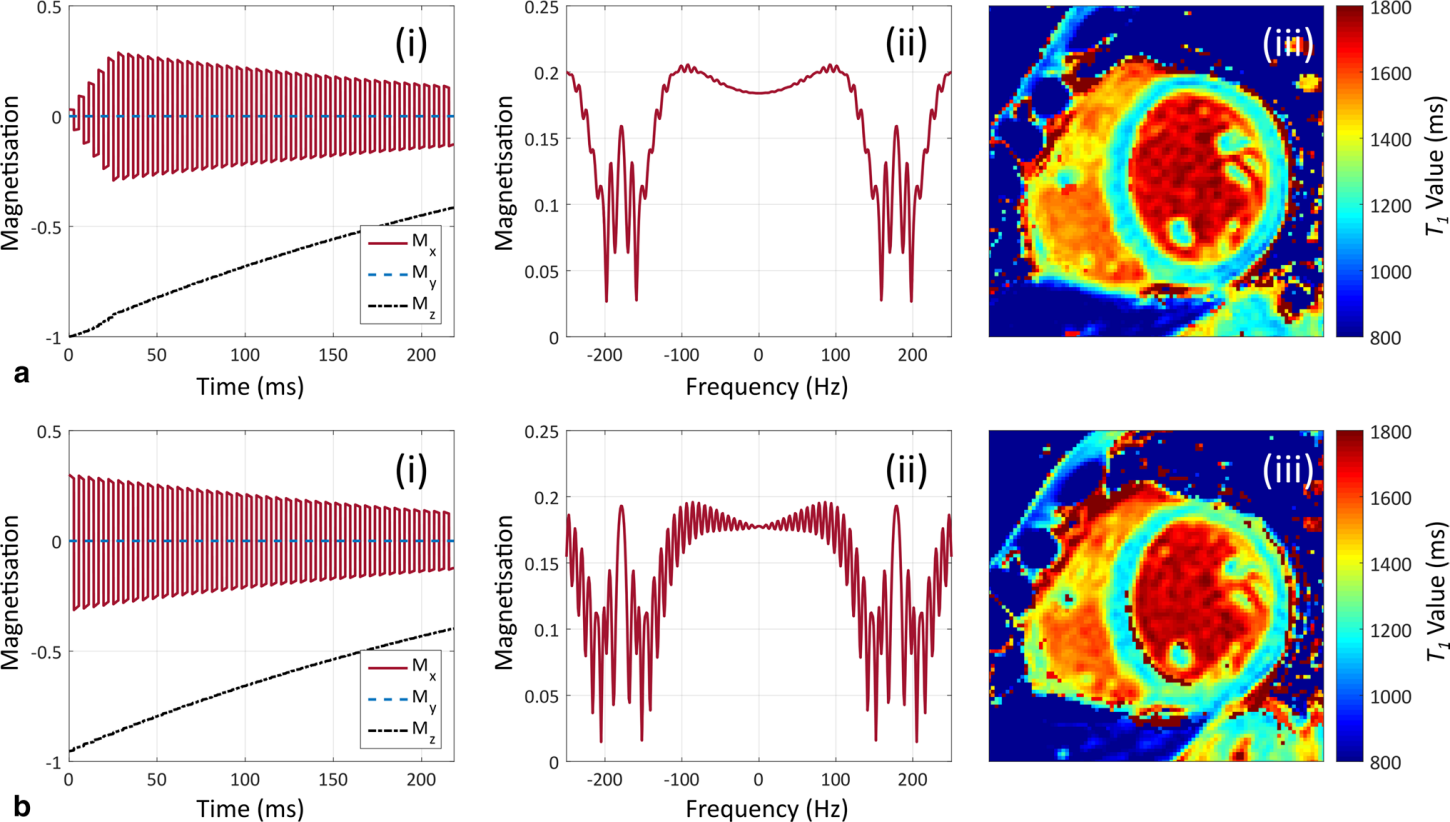
The effect of balanced steady-state free-precession catalysation sequences on native *T*_1_ maps. *Plots* show the effect of linear ramp-up (**a**) and half-alpha (**b**) catalysations on the magnetisation evolution (*i*) and frequency response at the centre of *k*-space (*ii*) of balanced steady-state free-precession at 3T. Short axis, mid-ventricular native *T*_1_ maps are shown for each method (*iii*). Simulations were performed with a repetition time of 2.8 ms, a flip angle of 35°, a *T*_1_/*T*_2_ of 1200/45 ms, and 10 catalysation pulses for each method, with a further 39 pulses before the centre of *k*-space

Several catalysation or “priming” sequences are used in bSSFP to accelerate the signal’s approach to the steady state, and these are particularly important in *T*_1_ mapping’s limited time window before image data must be acquired, especially for centre-out *k*-space ordering, which is discussed later. Catalysation details may be concealed from the scanner’s user interface, and may change through software upgrades without warning. Schemes include half-alpha [51], and linear ramp-up [52] (see Fig. 3); the type and duration of the scheme affects *T*_1_ estimation accuracy and precision, with bias errors of the order of 5% or more for some approaches [53].

The linear sweep up scheme is a good approach, as this sufficiently quells oscillatory behaviour prior to *k*-space filling [53].

#### Impact of *T*_2_ on estimation of *T*_1_ by bSSFP readouts

Shorter *T*_2_ relaxation times are associated with underestimation of *T*_1_ due to the *T*_2_/*T*_1_ weighting of bSSFP [5, 43], and their effect on preparation pulses. The Look–Locker sets of MOLLI sequences are more sensitive to *T*_2_ than the independent images used in saturation methods like SASHA, due to accumulated *T*_2_-related saturation between single-shot images in each set [54]. When *T*_2_ is long, such as in left ventricular blood, MOLLI estimation of *T*_1_ increases towards the true value.

In addition to low flip angles and short TR, coarser acquired resolution in tandem with parallel imaging and partial Fourier in the phase-encode direction can reduce the length of the bSSFP pulse train and thus mitigate *T*_1_ estimation bias resulting from *T*_2_-related saturation.

#### Impact of off-resonance on bSSFP readouts

A disadvantage of bSSFP is its sensitivity to off-resonance [55], which causes dark banding artefacts and associated *T*_1_ errors; however, off-resonance errors in estimated *T*_1_ can also occur in myocardial regions without banding artefacts. The inferolateral segment is particularly vulnerable to this problem. Furthermore, the off-resonance sensitivity of bSSFP is also influenced by the catalysation sequence used, as shown in Fig. 3.

The sensitivity to off-resonance is not identical across all of the bSSFP source images. Given that single-shot bSSFP is not fully stabilised for *T*_1_ mapping sequences, the impact of off-resonance varies at different points on the *T*_1_ recovery curve, and so does not cancel out of the curve-fitting estimation of *T*_1_ [48]. ECV measurements are less strongly affected by off-resonance than native *T*_1_ estimates, showing a bias error of around 1% or less [48].

Off-resonance errors can be reduced through volume *B*_0_ shimming over the heart and great vessels; however, even if second-order *B*_0_ shimming is carefully optimised, it cannot correct very local *B*_0_ distortions (Fig. 2a). Investigators should familiarise themselves with *B*_0_ shimming routines on their scanner, considering factors such as cardiac gating and respiration, among others. Projection-based shimming algorithms [56] are widely available; however, image-based shimming methods [57, 58] may offer better control over local *B*_0_, and they provide *B*_0_ fieldmaps that can assist quality control of *T*_1_ studies.

Off-resonance distortion of *T*_1_ can be reduced by lower flip-angles and/or a shorter TR. For example, a coarser frequency-encode resolution may reduce TR; however, this may also automatically modify the phase-encode resolution, reducing the number of RF pulses before the centre of *k*-space and affecting stabilisation. Furthermore, coarser frequency-encode spatial resolution increases partial volume. Any such changes require attention with regards to effects on estimated *T*_1_ and possible invalidation of normal range data [13].

A recent development substituted MOLLI’s bSSFP readout with a spoiled GRE sequence [59], which avoids the more complex sensitivities of bSSFP, improves *T*_1_ estimation accuracy, and reduces the sensitivity of *T*_1_ estimation to *T*_2_. It has also been applied in patients with implanted devices that cause severe off-resonance artefacts, precluding bSSFP imaging [60]. However, these benefits come with several disadvantages: spoiled GRE shows reduced SNR versus bSSFP; its *T*_1_ estimation precision is also reduced; and adequate spoiling may be difficult. Spoiling has been shown to be problematic for the variable flip angle method [61], but may be less so for inversion-recovery based *T*_1_ mapping [59].

### Schemes for *k*-space filling

To date, most *T*_1_ mapping has used linear phase-encode ordering, where phase-encoding gradient amplitudes are stepped through incrementally. This avoids eddy-current-related signal perturbations, but causes progressive *T*_2_-related saturation in the approach to the centre of *k*-space, leading to *T*_1_ underestimation. Alternatively, centric phase-encode ordering, also known as the centre-out or low–high approach, fills *k*-space from the centre outwards with alternating and increasing phase-encoding gradient amplitudes; this avoids the *T*_2_-related saturation of linear ordering at the expense of increased eddy-current-related artefacts [53].

Although linear-ordering is typically used, several other phase-encode ordering schemes have been investigated to date [43, 53, 62]. Paired phase-encoding has been proposed to mitigate the artefacts associated with centric ordering [63]; however, it has shown mixed results for *T*_1_ mapping [53, 62], performing well only with longer catalysation schemes.

### Signal-to-noise

All *T*_1_ mapping methods acquire multiple *T*_1_-weighted source images, each of which has its own SNR per tissue, and the noise level will influence the sampled points during curve-fitting. The fewer *T*_1_-weighted source images used to reconstruct a *T*_1_ map, the poorer the curve-fit conditioning and the poorer the *T*_1_ estimation precision [15]. Given the limited number of shots taken throughout longitudinal recovery, their optimum distribution in comparison to the relevant range of *T*_1_ is also important, motivating different sequence schemes for native and for post-contrast mapping [5, 27].

SNR varies spatially across the heart, predominantly due to the sensitivity profile of the receiver coil array. Low SNR is most evident in the lateral wall, which is farthest from the coil, and thus this region is more prone to noise-related *T*_1_ estimation bias and dispersion [4]. This effect is in addition to susceptibility artefact seen in the lateral wall—another reason why clinical *T*_1_ measurements for assessment of diffuse fibrosis are often confined to the interventricular septum [30].

Imaging at higher field strengths can mitigate errors resulting from low SNR, as shown by Piechnik et al. [15], who reported approximately 30% reduction in coefficients of variation for MOLLI and ShMOLLI *T*_1_ estimates when moving from 1.5 to 3T. Conversely, 3T exacerbates off-resonance and *B*_1_ inhomogeneity effects, though their impact can be controlled.

### Influence of field strength

In addition to the aforementioned off-resonance and *B*_1_ inhomogeneity issues at higher field strengths, and the potentially increased SNR, there are also differences in native *T*_1_ and *T*_2_ values between 1.5 and 3T.

A large multicentre study of native *T*_1_ and ECV values in normal myocardium, using original 3b(3b)3b(3b)5b MOLLI, reported mean (standard deviation) native *T*_1_ values of 950 (21) ms at 1.5T and 1052 (23) ms at 3T, and mean (standard deviation) ECVs of 0.25 (0.04) at 1.5T and 0.26 (0.04) at 3T [64]. The increased *T*_1_ at 3T can lead to insufficient longitudinal recovery between Look–Locker sets, causing *T*_1_ underestimation; furthermore, reduced myocardial *T*_2_ at 3T relative to 1.5T introduces additional negative bias due to the *T*_2_/*T*_1_ weighting of bSSFP and signal decay during preparation pulses [5].

Increased *B*_0_ and *B*_1_ inhomogeneity at 3T can be mitigated using appropriate *B*_0_ and RF shimming, respectively. Shallower excitation flip angles can also allay these effects, if the increased SNR at 3T is traded off [5].

### Breath-holding

**Fig. 4:**
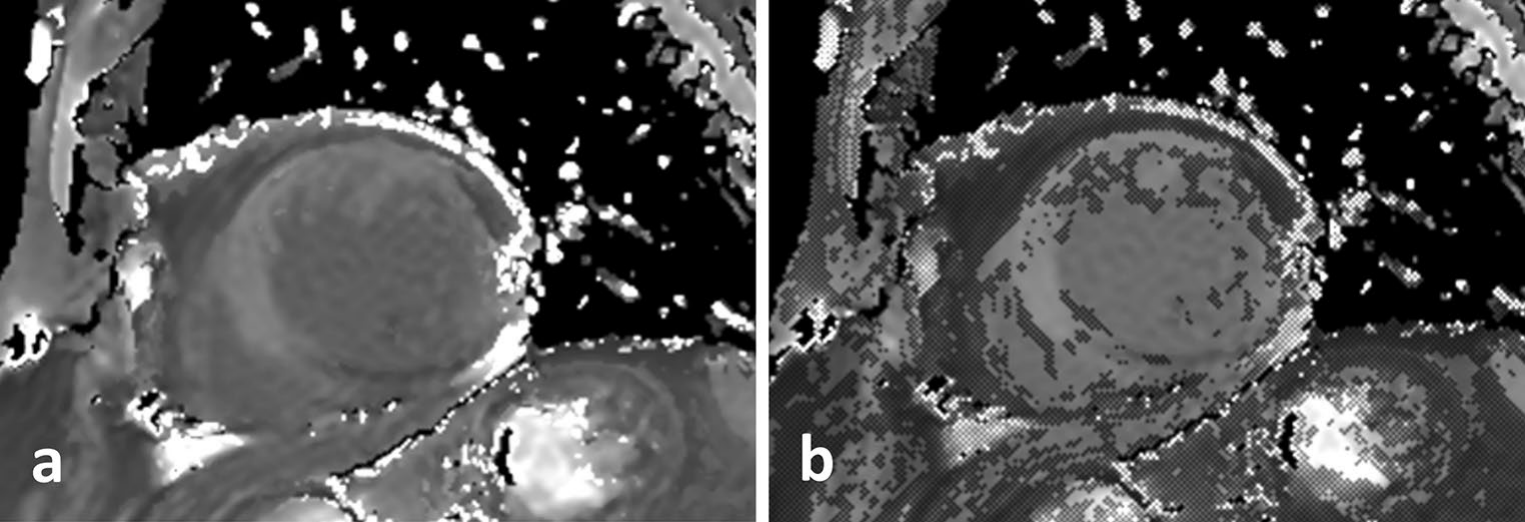
An example of motion-related *T*_1_ estimation error shown on quality control maps. A short-axis native *T*_1_ map corrupted by respiratory motion (**a**) demonstrates excessive pixelwise curve-fitting residual errors, indicated on a confidence map (**b**) by the addition of marked pixels to the same map shown in **a**

Currently, *T*_1_ mapping requires breath-holding to minimise respiratory motion while source images are acquired. Original MOLLI used a breath-hold duration of approximately 17 cardiac cycles [1], while newer variants require around 10 or 11 s [4]. A shorter breath-hold is an advantage in routine work [15], but it causes a reduction in SNR. Imperfect breath-holds typically lead to misregistered source images, which corrupt the set of signal intensities used for pixel-by-pixel curve fitting and, in turn, decrease *T*_1_ estimation accuracy and precision. Often, misregistration is not readily apparent in calculated *T*_1_ maps, unless a confidence-map is provided alongside or overlaid on the *T*_1_ map. See Fig. 4 for an example of this.

Post-acquisition quality control has some aspects in common for respiratory motion and cardiac misgating or arrhythmia: *T*_1_ mapping source images should be examined carefully for displacements, even if motion-corrected images are also available. Mislocated tissue in or through the selected slice, for any of the shots, may preclude correct mapping of localised disease, such as myocarditis. If significant displacements are found, nonrigid registration may register most myocardial pixels [20, 65], but cannot correct through-slice displacement. Input image registration in *T*_1_ mapping is challenging due to the large image-contrast variations between source images, and can be unreliable when tissues are imaged near the null point of longitudinal magnetisation recovery. While there are strategies for dealing with this issue [65], motion-corrected images should be reviewed before drawing ROIs on the *T*_1_ map. This issue is another reason why midwall, septum-only ROIs tend to be more reliable.

Free-breathing *T*_1_ mapping acquisitions have been reported [34, 36–39], and aim to automatically exclude images with large misregistrations. While this may be more feasible for independent images, as used in SASHA [16], the impact of poor breath-holding or misgating variations on the later points of a Look–Locker set is convoluted. Furthermore, these methods can extend scan time, and often employ undersampling.

### Cardiac triggering and cardiac motion

Source images for mapping must be acquired in the same phase of the cardiac cycle to ensure registration for pixelwise *T*_1_ map calculation.

Some variation in R–R interval is normal, and MOLLI-variant sequences record the real-time R–R increments to the inversion-recovery time during each Look–Locker set. Arrhythmia may be tolerable provided the trigger to the pulse sequence is followed by a reasonably normal ventricular contraction and diastolic pause (diastasis). If arrhythmia interferes with diastolic timing, it is feasible to acquire the single-shot images in end-systole [66, 67]. The end-systolic duration is less dependent on heart rate than diastasis, and may offer improved accuracy and precision in arrhythmia. Partial volume may also be less of an issue, as the contracted myocardium is thickened; however, the brevity of the end-systolic pause necessitates a shorter single-shot image readout, and thus spatial resolution is coarser.

A slightly shorter native *T*_1_ has been reported for mapping at end systole versus end diastole [66, 67]; however, this relationship flips after contrast administration, impacting ECV [68]. The cardiac phase of the image does not imply that the entire, usually slower, *T*_1_-relaxation process is sensitive to the myocardial relaxation or contraction state at the time of the image.

Similar to respiratory motion, cardiac mistriggering causes mis-registration of source images for *T*_1_ mapping. Again, an elastic image registration algorithm may be able to account for this, but source images should be checked rigorously. If mis-registration goes uncorrected, distortion of fitted *T*_1_ recovery curves is likely, particularly in the subendocardium. For this reason, some types of cardiac arrhythmia can be a major problem, but novel methods promise robust performance in such conditions [69].

The single-shot imaging duration should not exceed the length of the cardiac pause, be it diastolic or systolic. Tong et al. empirically estimated that the shot duration should not exceed 150 ms for minimal cardiac motion artefacts [70]. It is relatively straightforward to plan single-shot imaging to coincide with the required cardiac phase, as timings can be measured from a bSSFP cine acquired during routine setup, or may be semi-automated [71].

The image-readout duration can also be reduced using parallel imaging methods that acquire coil profiles separately: namely, before the scan or immediately after the last single-shot image. Note a related pitfall with coil profiles or any other prescan applied immediately prior to a *T*_1_ mapping acquisition is that the longitudinal magnetisation may not have recovered before the first inversion [48], though this is usually avoided with a pause for breath hold instruction to the patient.

### Flowing blood

Measurement of blood *T*_1_ is important for the calculation of ECV. Complications in Look–Locker correction arise for blood that is at least partially replaced by fresh wash-in in the image slice for each cardiac cycle. Completely “fresh” blood, while still acted upon by the initial non-selective inversion, has not experienced previous shots of the current Look–Locker set since inversion and, therefore, does not require Look–Locker correction. However, even a normal heart ejects only around 55–75% of left ventricular blood per cycle [72], so the true situation is probably a complex mixture of different magnetisation histories in left-ventricular blood. Furthermore, in the extreme case, for later images of a native *T*_1_ mapping acquisition the arriving blood may have experienced a distorted magnetisation preparation at some upstream location, despite application of optimised preparation pulses. This can occur in short bore scanner systems [5], or in unusual flow pathways following repairs of congenital heart defects.

### Magnetisation transfer (MT)

When estimating *T*_1_ in the myocardium and blood pool, it is also important to consider the MT phenomenon, which has been shown to influence the accuracy of *T*_1_ mapping [5, 47]. Exchange between free and bound water pools within the tissue of interest reduces the bSSFP signal [73]. For inversion-recovery-based *T*_1_ mapping, the bound pool is mostly unaffected by the inversion pulse, so exchange during the long inversion-recovery delays distorts the shape of the *T*_1_ recovery curve and introduces *T*_1_ underestimation. The extent of this effect varies between tissues, and is substantially smaller in blood than in myocardium [5, 74].

The MT effect can be allayed by using SASHA with a three-parameter curve-fit, at the expense of reduced *T*_1_ estimation precision. It can also be mitigated in inversion-recovery *T*_1_ mapping by use of lower-flip-angle excitation pulses and a longer imaging TR. Alternatively, the MT effect could be exploited in native *T*_1_ mapping for greater disease discrimination in pathologies such as myocardial infarction (MI), ischaemia, and iron overload—all of which have all demonstrated MT.

### Summary of technical pitfalls

Each technical and physiological challenge listed here may cause errors in *T*_1_ and ECV mapping, increasing bias, scatter, or both. Cardiac and respiratory motion are particularly problematic, and strict quality control routines may help correct these where possible. At 3T, inhomogeneous *B*_0_ and RF transmit fields also become prominent sources of error, which may be mitigated with appropriate *B*_0_ shimming and RF transmit calibration. However, despite these prominent pitfalls, no issue dominates, and with considerable expertise, care, and attention to multiple factors, users can mitigate many sources of error, with the level of optimisation depending on their specific clinical or research applications.

## Contrast agents

Gadolinium-based contrast agents (GBCAs) in myocardial *T*_1_ mapping are subject to their own issues and controversies for deriving estimates of myocardial ECV. This section discusses the various assumptions made about GBCAs in *T*_1_ and ECV mapping.

### Assumptions relating to contrast agents

Several basic assumptions regarding GBCA estimation of ECV are stated here first, with further details later. Strictly, we assume that the contrast agent has identical relaxivities, *r*_1_, in myocardium and blood pool:

3 $$\Delta R_{{1,{\text{myo}}}} = r_{{1,{\text{myo}}}} \left[ {\text{Gd}} \right]_{\text{myo}}$$

4 $$\Delta R_{{ 1 , {\text{blood}}}} = r_{{1,{\text{blood}}}} \left[ {\text{Gd}} \right]_{\text{blood}} ,$$

where $\Delta R_{{1,{\text{myo}}}}$ and $\Delta R_{{1,{\text{blood}}}}$ are the changes in relaxation rates in myocardium and blood, respectively, and $[ {\text{Gd]}}$ represents the concentration of GBCA, typically in millimole/litre units [75, 76]. The change in relaxation rate, $\Delta R_{1}$, is given as:

5 $$\Delta R_{1} = \left( {{1 \mathord{\left/ {\vphantom {1 {T_{1} }}} \right. \kern-0pt} {T_{1} }}} \right)_{\text{postGad}} - \left( {{1 \mathord{\left/ {\vphantom {1 {T_{1} }}} \right. \kern-0pt} {T_{1} }}} \right)_{\text{native}} .$$

If we assume that $r_{{ 1 , {\text{myo}}}} = r_{{1,{\text{blood}}}}$, as stated above, then:

6 $$\Delta R_{1} = r_{1} \left[ {\text{Gd}} \right] \to {{\Delta R_{{1,{\text{myo}}}} } \mathord{\left/ {\vphantom {{\Delta R_{{1,{\text{myo}}}} } {\Delta R_{{1,{\text{blood}}}} }}} \right. \kern-0pt} {\Delta R_{{1,{\text{blood}}}} }} = {{\left[ {\text{Gd}} \right]_{\text{myo}} } \mathord{\left/ {\vphantom {{\left[ {\text{Gd}} \right]_{\text{myo}} } {\left[ {\text{Gd}} \right]_{\text{blood}} }}} \right. \kern-0pt} {\left[ {\text{Gd}} \right]_{\text{blood}} }},$$

where the ratio ${{\left[ {\text{Gd}} \right]_{\text{myo}} } \mathord{\left/ {\vphantom {{\left[ {\text{Gd}} \right]_{\text{myo}} } {\left[ {\text{Gd}} \right]_{\text{blood}} }}} \right. \kern-0pt} {\left[ {\text{Gd}} \right]_{\text{blood}} }}$ is the partition coefficient, *λ* [75, 77].

Secondly, we assume that the GBCA does not enter myocytes or blood cells, and that it is instead in dynamic equilibrium of water relaxation inside those cells, because of fast-exchange of water through cell walls, as follows:

7 $$\left[ {\text{Gd}} \right]_{\text{myo}} = \left[ {\text{Gd}} \right]_{\text{interstitial}} \times {\text{ECV}}$$

8 $$\left[ {\text{Gd}} \right]_{\text{blood}} = \left[ {\text{Gd}} \right]_{{{\text{bl\_plasma}}}} \times \left( {1 - {\text{Hct}}} \right),$$

where Hct is the haematocrit. The term “fast” implies fast exchange of enough water across cell walls relative to the relevant *T*_1_ range late after the myocardial first-pass.

Both amyloid deposition and collagen accumulation in fibrosis increase the interstitial space and break up myocyte packing. Collagen itself is of negligible non-permeated or “dark” volume (very short *T*_2_), and is assumed to be highly permeable to interstitial fluid, including the GBCA. If the GBCA did not enter the collagen volume, but achieved relaxation equilibrium with it by fast exchange, it would manifest as an abnormally low ECV, resembling myocyte hypertrophy.

Finally, late after injection we assume that the concentration of GBCA in the interstitial fluid is equal to that in the blood plasma [77], and we calculate ECV as follows:

9 $$\left[ {\text{Gd}} \right]_{\text{interstitial}} = \left[ {\text{Gd}} \right]_{{{\text{bl\_plasma}}}} \to {\text{ECV = }}\left( {1 - {\text{Hct}}} \right) \times {{\Delta R_{{1,{\text{myo}}}} } \mathord{\left/ {\vphantom {{\Delta R_{{1,{\text{myo}}}} } {\Delta R_{{1,{\text{blood}}}} }}} \right. \kern-0pt} {\Delta R_{{1,{\text{blood}}}} }},$$

as described by Messroghli et al. [78].

### Contrast agent types

Several GBCAs are available for *T*_1_ mapping applications, including gadopentetate dimeglumine (Gd-DTPA), gadobenate dimeglumine (Gd-BOPTA), and gadobutrol; these are known under the trade names “Magnevist”, “Multihance”, and “Gadovist”, respectively. Each agent has differing relaxivities and binding properties, which can lead to differences in the estimated ECV; further complications arise due to relaxivity variations with different field strengths.

The Gd-BOPTA agent’s aromatic ring enables weak plasma protein binding, leading to a lower molecular tumbling rate and thus a longer rotational MR correlation time and higher relaxivity in blood plasma and myocardial interstitial fluid compared to Gd-DTPA and gadobutrol. Furthermore, a lower dose of Gd-BOPTA has been shown to have similar diagnostic efficacy to a higher dose of Gd-DTPA in LGE imaging of MI [79]. Kawel et al. have shown that the use of Gd-DTPA leads to myocardial *T*_1_ values around 15 ms lower than Gd-BOPTA, with no statistically significant difference seen in blood pool [80]. This results in slightly greater ECV values measured by Gd-DTPA, of the order of 0.01, perhaps due to Gd-BOPTA’s binding to human serum albumin, which is responsible for its increased relaxivity. With regards to relaxivity variations with field strength, work by Rohrer et al. and Pintaske et al. has illustrated the variability in *R*_1_ of GBCAs in human blood plasma for different contrast agent types and at different field strengths [81, 82], which will lead to bias and variability in ECV measurements if not accounted for.

The relatively greater presence of albumin in blood versus myocardium means the distribution of protein-bound contrast agent between these pools is likely to be different than for non-protein-bound equivalents, altering the ratio of the change in relaxation rate of myocardium and blood and thus altering ECV. Given that this distorts one of the assumptions of in gadolinium-based ECV estimation, use of a protein-bound contrast agent will slightly modify partition coefficient estimation by *T*_1_ mapping. If investigators plan to use one of these agents, they should do so consistently, and report this clearly in any inter-site comparisons.

### Steady-state contrast versus bolus administration

**Fig. 5:**
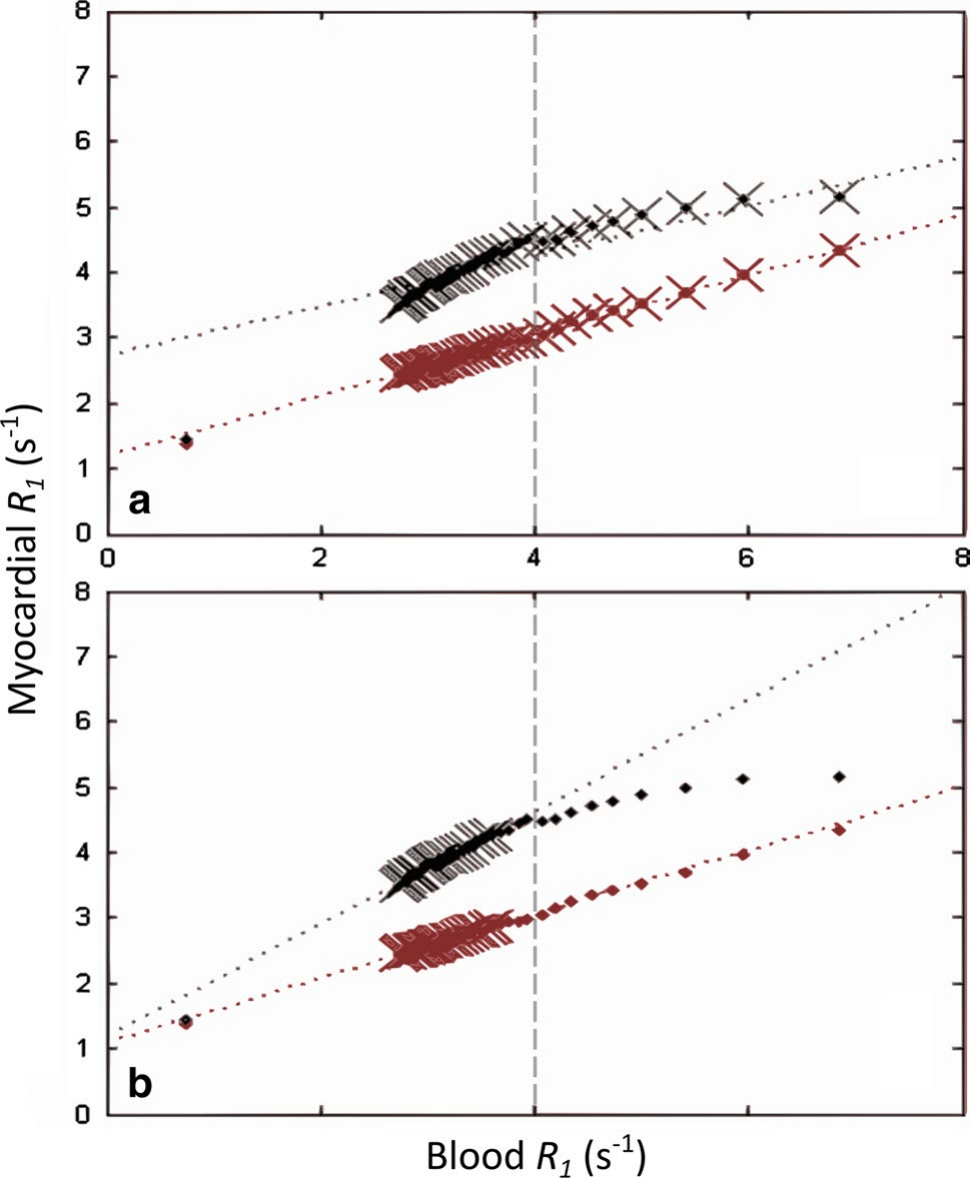
Estimation of partition coefficient in chronic myocardial infarction patients. *Plots* show myocardial relaxation rate (*R*_1_) versus blood *R*_1_ in viable and infarcted myocardium (*red* and *black points*, respectively) for two different post-contrast serial acquisition schemes. Note that the slopes of the linear regressions (partition coefficients, *λ*, indicated by *dotted lines*) change considerably depending on the sampled points (*crosses*) for infarcted tissue, but remain relatively constant in viable myocardium. *Plot* (**a**) used time points from 1 to 40 min for partition coefficient estimation, which gave *λ* = 0.46 in viable myocardium and 0.38 in infarct. *Plot* (**b**) used time points from 15 to 40 min for linear regression, leading to *λ* = 0.49 in viable tissue and 0.86 in infracted tissue. *Dashed grey lines* indicate the cut-off *R*_1_ of 4 s^−1^, above which points are generally excluded to avoid nonlinearity of *λ* (Jerosch-Herold, personal communication). Adapted with permission from Goldfarb and Zhao [90]

The method of contrast administration also introduces variability to ECV calculation. Under most conditions, the two-compartment steady-state assumption, stated in Eq. (9), holds true for single bolus administration [83–85]. Early work in ECV used a primed infusion approach, whereby an initial loading bolus is followed by a continuous infusion of GBCA [86]. For most situations, the simpler bolus-only approach gives a similar ECV; however, for ECV greater than 0.4, in myocardial infarction (MI) and amyloidosis for example [87], it substantially overestimates ECV [84]. In a third method, several *T*_1_ mapping acquisitions can be acquired during GBCA washout for improved accuracy in estimating the gadolinium blood-myocardium partition coefficient [24, 88, 89], which is estimated through the slope of a linear fit to myocardial *R*_1_ versus blood *R*_1_. The partition coefficient has been shown to deviate from this model in the early washout phase [90, 91], causing underestimation of ECV. This can be seen in Fig. 5, where data become markedly non-linear for blood *R*_1_ values greater than 4 s^−1^; these data are often excluded from ECV calculations to avoid bias (Jerosch-Herold, personal communication).

### Fast exchange assumption

Equations (7) and (8) are dependent on the fast exchange assumption, whereby we assume that water exchange between intracellular and extracellular compartments is sufficiently fast relative to the difference between the relaxation rates of the compartments considered in isolation [75]. In cases where this assumption is broken, where higher GBCA concentrations are used or post-GBCA measurements are made too early, as discussed above, ECV may be underestimated [92]. Regardless, in typical clinical *T*_1_-mapping situations, it appears that the fast-exchange assumption is sound.

### Use of blood *T*_1_ to calculate haematocrit

The blood for haematocrit assessment should be taken contemporaneously with *T*_1_ mapping [13], to avoid unnecessary scatter in ECV. Synthetic haematocrit has recently been proposed as a means of streamlining ECV calculation [93]; it is calculated using the linear relationship between native blood *T*_1_ and blood-analysed haematocrit. Support for this approach is spreading [93, 94]; however, several issues have been identified that should be considered [95, 96].

### Summary of contrast agent issues

Although there are multiple issues with contrast agent types and field-strengths, none of these appear to dominate. There perhaps remains a dilemma between optimal ECV assessment and clinical feasibility: for example, the use of multiple acquisitions during GBCA washout, along with multiple averages for native *T*_1_ scans, will improve ECV accuracy and precision, but such a protocol is difficult to fit into busy clinical schedules.

## Errors in specific clinical applications

The aim of this section is to highlight the impact of the above topics in specific clinical applications, with examples and possible solutions.

### Differences in age, sex, and myocardial region

There appear to be subtle differences in native myocardial *T*_1_ related to sex and age, though there is currently no consensus on whether these also influence ECV [28, 64, 97–99]. Several investigators have posited theories as to why native *T*_1_ and ECV might increase or decrease with age, but the debate over these issues is outside the scope of this review. We should, however, point out that these changes are small, and demonstrable only over large groups, with a similar scatter to *T*_1_ and ECV estimates in diffuse fibrosis, which are discussed later.

**Fig. 6:**
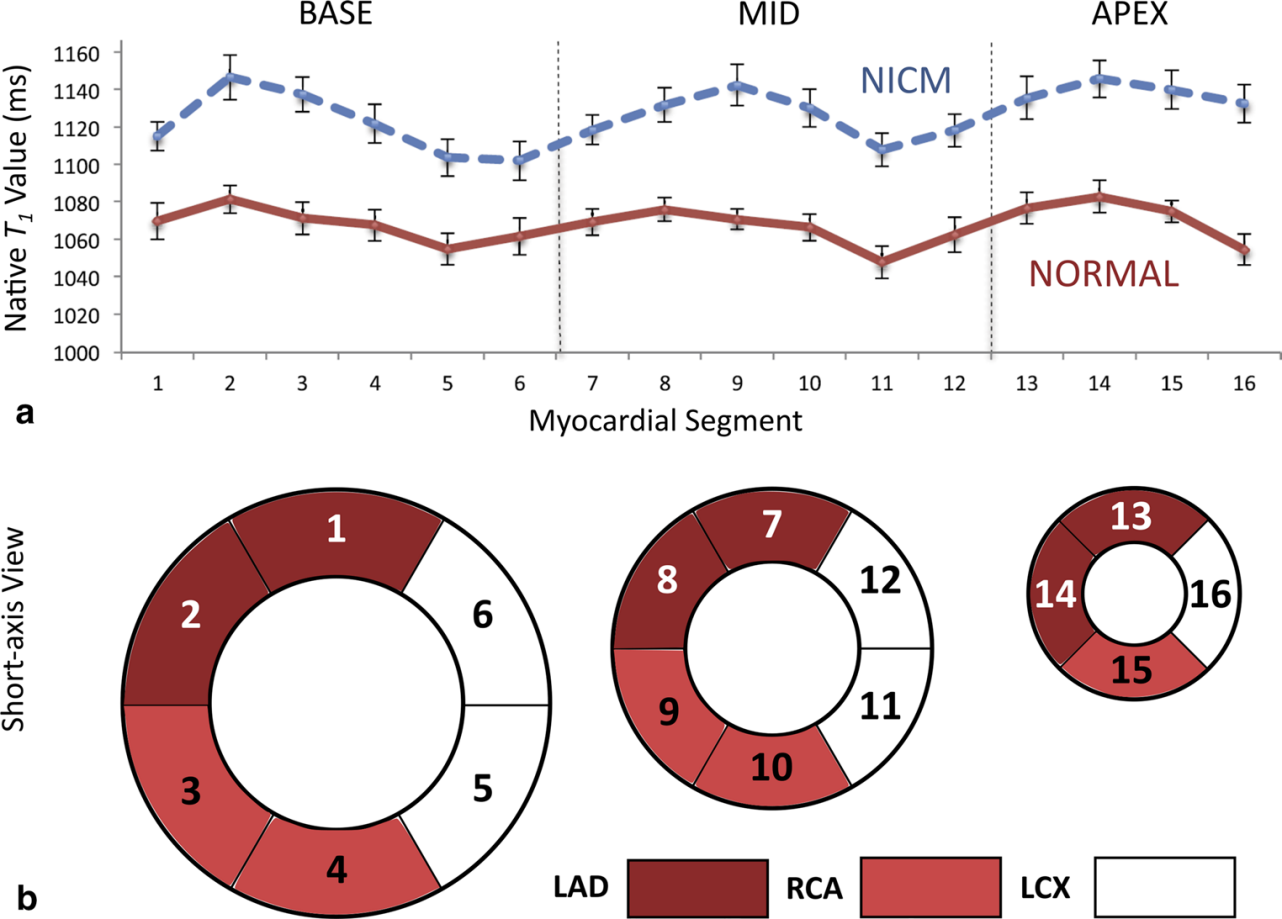
Regional variation in native *T*_1_ estimates. Graph (**a**) shows variability in segmental native *T*_1_ across 16 myocardial segments in subjects with normal left ventricular function (*solid red line*, *n* = 27) versus subjects with non-ischemic cardiomyopathy (*dashed blue line*, *n* = 39). *Error bars* represent the standard error of the mean. Base, mid, and apex refer to levels of the left ventricular myocardium, and segments 2, 3, 8, 9, and 14 are septal, as illustrated by the American Heart Association model (**b**). Inferolateral segments show the lowest native *T*_1_ values, likely due to off-resonance at interfaces with the lung and the posterior vein of the left ventricle. The approximate territories of the right coronary artery (RCA) and the left anterior descending (LAD) and left circumflex (LCX) arteries are also shown for interest. Graph (**a**) is reproduced with permission from Shah et al. (2016) Am J Cardiol 117(2): 282–288

Regarding myocardial region, there appears to be no statistically significant difference between native *T*_1_ measurements in the basal, mid, and apical regions of the left ventricle in healthy volunteers [100]. Several studies, however, have reported lower native *T*_1_ values in the lateral wall versus the inter-ventricular septum [30, 64, 68, 97, 101]. It is likely this is mainly due to technical confounds rather than physiological differences, as cardiac motion, off-resonance by local *B*_0_ distortion, and lower coil sensitivity all reduce accuracy and precision in the lateral wall. ECV, on the other hand, does not differ significantly between the lateral wall and septum [97], suggesting that reduction of native *T*_1_ by off-resonance is likely the main source. Figure 6 illustrates the variation of native *T*_1_ throughout the heart; lower *T*_1_ values are seen in inferolateral segments, which typically demonstrate off-resonance due to interfaces with the lung and the posterior vein of the left ventricle [102, 103].

### Routine clinical applications of *T*_1_ and ECV mapping

Myocardial *T*_1_ and ECV mapping has attracted a lot of interest for both clinical and research applications due to its potential for accurate and precise tissue characterisation on a pixel-by-pixel basis. While *T*_1_ mapping still shows promise for aiding precision medicine and influencing management of individual patients, recent work has been more pragmatic, with applications focusing on large patient populations and specific conditions.

#### Cardiac amyloidosis

Amyloid is a relatively rare multi-system condition where deposition of misfolded fibrillary protein in tissues and organs can cause expansion of the myocardial extracellular space and impairment of cardiac function [104]. This strongly increases native *T*_1_ and ECV globally, with minimal overlap with healthy ranges [84], making them an excellent diagnostic tool [105]. Post-contrast *T*_1_ mapping can also be helpful in highlighting abnormal GBCA washout kinetics, which show a specific pattern in amyloid patients [106], and a higher ECV in this context indicates a worse prognosis [107]. With large global changes and no concerns regarding myocardial region, this combination enables *T*_1_ mapping to deliver strong diagnostic and prognostic utility.

#### Anderson-Fabry disease

Another rare multi-system syndrome, Anderson-Fabry disease (AFD) is characterised by intracellular accumulation of glycosphingolipids. This leads to progressive cardiac, renal, and cerebrovascular disease [108], and thus early diagnosis is extremely important for timely intervention. AFD markedly reduces global myocardial native *T*_1_ compared to healthy volunteers, and thus represents a strong application of native *T*_1_ mapping [109–111], again without concerns about regional myocardial differences. For several reasons, the reduced global native *T*_1_ in AFD likely does not result directly from the short *T*_1_ of fat [109], contrasting with apparent local *T*_1_ increases sometimes seen in fatty infiltration of chronic MI, which is discussed later.

#### Myocarditis and Takotsubo cardiomyopathy

Myocarditis and Takotsubo cardiomyopathy are also potential clinical applications for *T*_1_ mapping [112, 113], being characterised by myocardial oedema, among several other markers. Oedema can be clearly highlighted on native *T*_1_ maps due to increased interstitial fluid content; indeed, native *T*_1_ mapping has higher diagnostic accuracy for identifying oedema than *T*_2_-weighted imaging in Takotsubo [113, 114], and myocarditis [115]. In this application, although native *T*_1_ is strongly increased, this is often sharply localised in myocardium, and thus operators should take care to localise to the relevant myocardial region. Furthermore, understanding of localised technical pitfalls is also valuable: such as off-resonance, whose impact is further modulated by *B*_1_ changes over the heart. It should be noted that published studies have typically excluded patients with major epicardial coronary disease or past MI. For *T*_1_ mapping to become clinically meaningful in myocarditis and Takotsubo, it should not only positively confirm the diagnosis, but also rule out acute MI.

### Potential clinical applications of *T*_1_ mapping

In some pathologies, *T*_1_ and ECV currently demonstrate limited sensitivity, but may ultimately be of clinical utility if their precision is improved, or if confounds are addressed.

#### Acute myocardial infarction

Like myocarditis and Takotsubo, acute MI also presents with oedema, which leads to elevated local *T*_1_ on native *T*_1_ maps [116–118]. There are, however, potential *T*_1_ mapping pitfalls in acute MI, additional to that of local disease. Firstly, microvascular obstruction, or the “no reflow” phenomenon [119], causes derangement of the microvasculature, limiting blood flow post-reperfusion. This can distort an earlier assumption for ECV derivation, that of GBCA equilibrium between the infarct zone and the blood plasma. In particular, the necrotic core of the infarct will not be in equilibrium 15–20 min post-bolus, requiring infusion for accurate ECV measurement [84].

Secondly, myocardial haemorrhage often occurs concomitantly with microvascular obstruction [120], and is characterised by extravasation of red blood cells through gaps in the endothelial walls. This leads to a cascade of haemoglobin decay products in the no reflow region during the weeks following reperfusion, with various iron states affecting *T*_2_, estimated *T*_1_, and true *T*_1_ [121]. Clearly this poses problems for *T*_1_ mapping, and thus CMR studies should be timed appropriately after reperfusion therapy [122].

#### Chronic myocardial infarction

For *T*_1_ mapping, chronic MI presents the challenge of lipomatous metaplasia, which affects around 24–47% of MI patients [29, 123]. This is characterised by fatty infiltration of myocardium, highlighting the following technical difficulty with fat partial volume in *T*_1_ mapping.

**Fig. 7:**
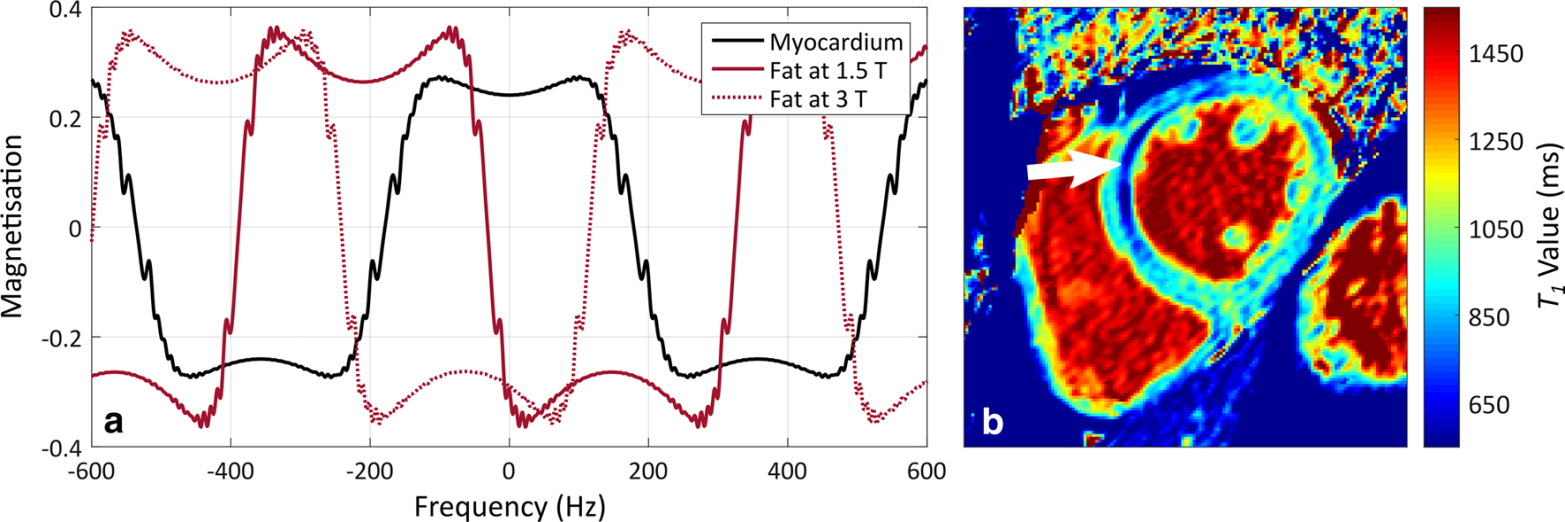
The effect of intramyocardial lipids on native *T*_1_ estimation in chronic myocardial infarction. The balanced steady-state free-precession off-resonance response is shown for myocardium and fat (**a**), for a modified Look–Locker inversion recovery protocol with a repetition time of 2.8 ms and a flip-angle of 35°. When fat and water are out of phase, lipids typically show an elevated *T*_1_ in *T*_1_ maps; when they are in-phase, lipid *T*_1_ typically appears *lower* than myocardial *T*_1_. An example native *T*_1_ map (**b**), acquired at 1.5T in a chronic myocardial infarction patient with lipomatous metaplasia, shows a reduced apparent *T*_1_ associated with lipid signals (*white arrow*). Both the accuracy and precision of native *T*_1_ estimates are influenced by this effect. Figure (**b**) was provided courtesy of Dr Heerajnarain Bulluck, The Hatter Cardiovascular Institute, University College London

Ignoring bSSFP characteristics, a mixture of in-phase lipid and water signals within a voxel produces a biexponential *T*_1_ recovery curve. Attempting to fit these data with a monoexponential curve typically leads to lower myocardial *T*_1_ estimates due to inclusion of short *T*_1_ lipids, which have *T*_1_ values around 370 ms at 1.5T and 450 ms at 3T [124]. However, in myocardial *T*_1_ mapping the mono- versus bi-exponential “model mismatch” issue is usually not the dominant factor, because most *T*_1_ mapping uses bSSFP, in which fat and water are typically out of phase due to the frequency offset of fat [125]. Complex interference between fat and water signals leads to counterintuitive results: for smaller fat fractions, around 0.5–40% [29, 126], an increase in the apparent myocardial *T*_1_ occurs; higher fat fractions lead to undefined *T*_1_ estimates; and fat-like *T*_1_ values are seen only at the highest fat fractions. In chronic MI, fat fractions typically do not exceed 35% [29], and thus a positive *T*_1_ estimation bias is expected, assuming fat and water are out of phase. This reduces the specificity of native *T*_1_ mapping in chronic MI, because similar, but genuine, changes occur in oedema or inflammation. It is currently unclear what effect fatty changes have on ECV. See Fig. 7 for a plot of water and fat signals in bSSFP, along with a native *T*_1_ map acquired in a chronic MI patient with lipomatous metaplasia.

The complex impact of fat partial volume in *T*_1_ mapping might only reliably be reduced by replacing bSSFP with spoiled GRE imaging, in which the fat phase-difference depends purely on the TE. However, the reduced SNR of spoiled GRE relative to bSSFP would have to be considered. Alternatively, quantitative myocardial fat-fraction mapping could be applied [127], as recent work has incorporated fat–water separation into MOLLI and SASHA *T*_1_ mapping in skeletal muscle [128] and the heart [32].

#### Diffuse myocardial fibrosis

Several pathologies cause diffuse fibrosis of the myocardium, such as hypertrophic and dilated cardiomyopathies (HCM and DCM), atrial fibrillation, aortic stenosis, heart failure with reduced or preserved ejection fractions (HFrEF and HFpEF), congenital heart disease, hypertension [129], and diabetes [130]. Certain drug therapies, such as alkylating agents in chemotherapy, can also lead to diffuse fibrosis [131], and possibly benefit from applications of *T*_1_ mapping [132].

**Fig. 8:**
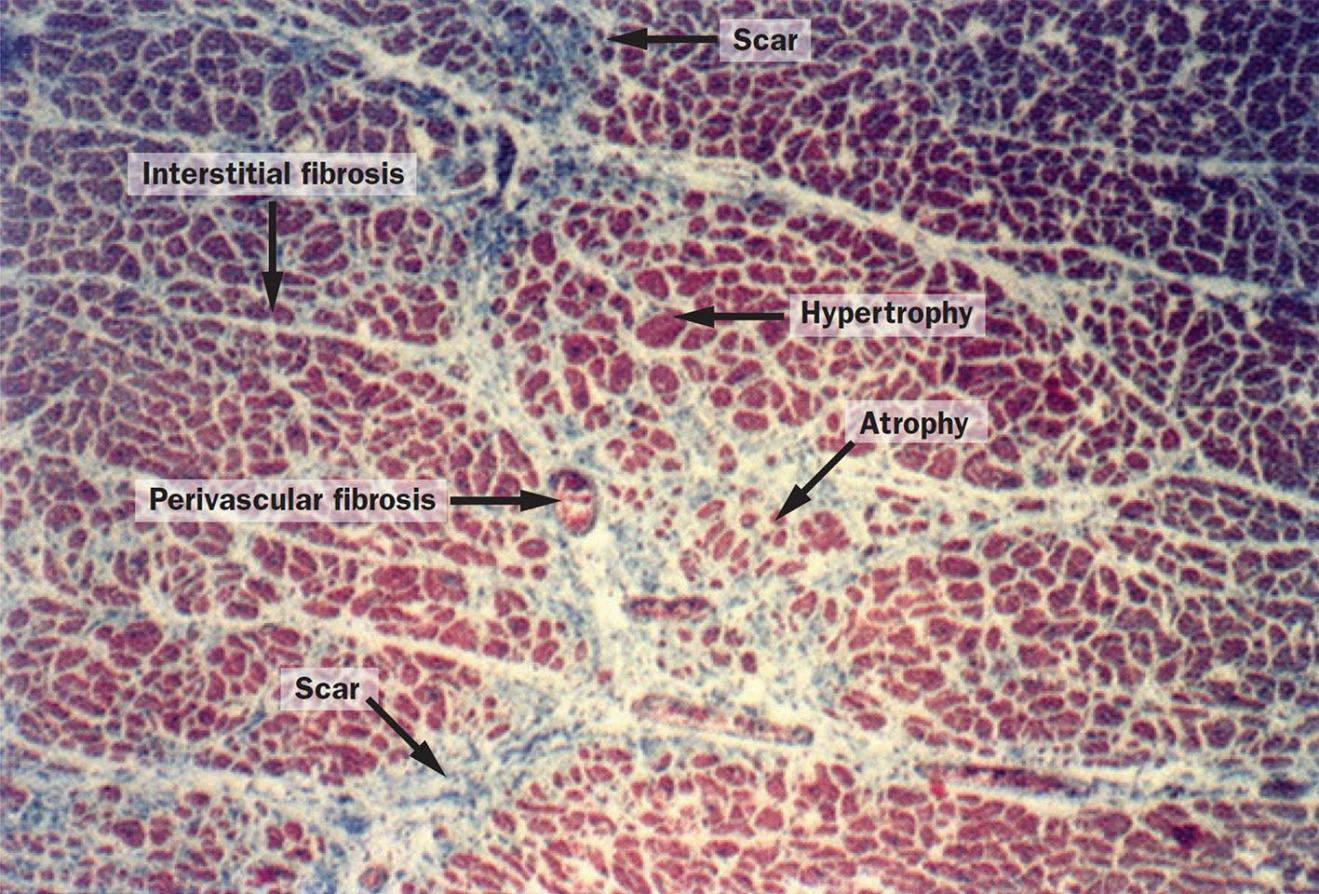
Structural remodelling of myocardium in hypertension. Subvoxel heterogeneity can be seen in cardiomyocyte size, which ranges from hypertrophied to atrophied, and in fibrosis, which consists of microscopic scars, and perivascular and interstitial fibrosis. Reproduced with permission from Weber et al. [136]

In general, native *T*_1_ values are increased in diffuse fibrosis, but not to as great an extent as in oedema or amyloidosis, and ECV is also slightly elevated. In general, scatter in estimated *T*_1_ values seems to be of a similar order to that seen in diffuse-fibrosis changes, and this has delayed the wider uptake of *T*_1_ mapping. Several investigators have shown the usefulness of ECV as a prognostic indicator [133, 134], but there seems to be little progress in taking this towards a per-patient test. In many applications there is a mixture of focal and diffuse fibrosis, which begs the question whether visible focal fibrosis was excluded from ROIs used in diffuse fibrosis studies [135], and even if so, what “mesoscopic” or “microscar” subvoxel focal fibrosis, or other myocardial changes, might be included in the so-called diffuse fibrosis assessment by *T*_1_ [136] (Fig. 8).

Clearly there is substantial overlap between native *T*_1_ and ECV values measured in controls versus those measured in patients with likely diffuse fibrosis. This is partly due to the small changes seen in diffuse fibrosis, particularly early in the disease when reversing it would be of great clinical benefit before irreversible damage occurs to the myocardium. It is also related to the many sources of dispersion in *T*_1_ parameter estimation. As yet, it appears that there is no one factor that can be adjusted to achieve adequate *T*_1_ estimation precision for detecting early diffuse fibrosis. Many recently reported clinical studies naturally retained older *T*_1_ mapping protocols, due to the constraints of their study length, or follow-up periods in the “prognosis” papers. Despite these problems on the individual level, which may be overcome by stricter control of errors, *T*_1_ mapping of diffuse fibrosis offers concrete benefits in large-scale studies: offering a means of testing treatment effects and characterising differences on a population level [130, 137].

### Summary of clinical sources of error

Both native *T*_1_ and ECV are important clinical measures that allow us to characterise the myocardium in a fashion complementary to LGE. The substantial overlap of these measures between patients and controls in some cardiac conditions offers challenges to the clinical use of *T*_1_ mapping, at least with current methodology. However, in specific conditions, such as amyloid and Anderson-Fabry disease, *T*_1_ mapping has an important diagnostic and prognostic role, and is being widely adopted into routine clinical use.

## Future directions of *T*_1_ and ECV mapping

Potential future solutions for errors in *T*_1_ and ECV mapping have been discussed throughout this review. We will now highlight several promising areas of development that may determine the future directions of the field.

Free-breathing *T*_1_ mapping would appear to offer major gains with regards to *T*_1_ mapping accuracy and precision, as well as enabling scanning of patients with compromised breath-holding. There are already several publications demonstrating the benefits of free-breathing *T*_1_ mapping [36–39]; however, the added time required for these methods will limit their wider uptake, unless retrospective image registration can be robustly applied.

Certain recent implementations of myocardial *T*_1_ mapping have incorporated simulations to improve the accuracy of bSSFP MOLLI [41, 138, 139], to enable spoiled GRE *T*_1_ mapping [59], and to reduce the number of pause intervals between Look–Locker sets [140]. As yet, these methods do not offer an advantage in precision over the original MOLLI implementation, but further work may demonstrate benefits to their use [138].

Simultaneous *T*_1_, *T*_2_, and proton density mapping of the myocardium is also possible [141–144], with some methods being feasible in a single breath-hold [142–144]. Magnetic resonance fingerprinting is an extension of simulation-based methods that can offer *T*_1_, *T*_2_, and proton density maps [145], as well as other parameters modelled in the dictionary. It has recently been adapted to the heart [146]; however, further work is required, as currently it shows inferior precision to conventional *T*_1_ mapping and requires long computation times for pattern matching.

## Sacrificing *T*_1_ accuracy for increased clinical utility

Placing a particular *T*_1_ estimate in a local normal range may necessitate high precision, but not high accuracy. Indeed, accuracy may be sacrificed deliberately to yield *T*_1_ estimates that are better able to discriminate normal tissue from pathology. For example, increasing the MOLLI excitation flip angle to 50° increases sensitivity to off-resonance, which reduces *T*_1_ accuracy, but also increases MT effects, which differ between tissue types. This appears effective in detecting diffuse fibrosis in the septum [30, 147]. The accuracy of this approach is limited, but the “true” *T*_1_ is less relevant if local normal ranges are used, as they should be for any *T*_1_ mapping implementation, under current guidelines [13].

Conversely, accuracy is important for establishing myocardial *T*_1_ and ECV as clinical biomarkers using normal ranges that are transferable between sites and vendors [148].

## Summary

This review has discussed reasons for inaccuracy and imprecision in *T*_1_ and ECV mapping, and it should be clear from these considerations that users should take great care when deviating from manufacturers’ advised *T*_1_ mapping protocols. While improving the apparent quality of maps and source images, users may inadvertently reduce the precision of *T*_1_ estimates, damaging clinical utility. Whatever *T*_1_ mapping setup is used, it is essential that its performance is characterised in local normal ranges, and that it is applied only for those clinical questions that its precision can support. Ongoing quality control and reassessment is also required to ensure a local normal range remains valid; the reader is referred to the upcoming SCMR and EACVI consensus for recommendations in this regard.

In the research and clinical applications described here, current native *T*_1_ and ECV mapping methods show utility in groupwise comparisons through to individual clinical tests. For some conditions, like diffuse fibrosis, mapping methods serve as weakly prognostic biomarkers that might be beneficial in combination with other diagnostic information about an individual patient. In other, albeit quite rare, conditions native *T*_1_ and ECV mapping can provide strong diagnostic data.

Fundamentally, cardiac mapping methods have not changed radically since Messroghli et al. first introduced MOLLI [1]; however, their diversity offers challenges to inter-centre use, and new developments and quality controls are still evolving. New approaches that incorporate various forms of undersampling and modelling, such as fingerprinting, do not currently offer substantial gains in accuracy and precision, other than avoiding dependence on breath-holding in some cases.

It remains unclear how much of the scatter observed in *T*_1_ estimates is due to physiological differences in true *T*_1_, or how much might be eliminated if the potentially correctable issues discussed here could be addressed robustly. If these pitfalls can be accounted for simply, quickly, and reliably, without need for specialist attention, *T*_1_ and ECV mapping may ultimately support more widespread clinical applications.
